# Agent-based simulation of collective cooperation: from experiment to model

**DOI:** 10.1098/rsif.2020.0396

**Published:** 2020-10-07

**Authors:** Benedikt Kleinmeier, Gerta Köster, John Drury

**Affiliations:** 1 Munich University of Applied Sciences, Department of Computer Science and Mathematics, 80335 Munich, Germany; 2 Technical University of Munich, Department of Informatics, 85748 Garching, Germany; 3 University of Sussex, School of Psychology, BN1 9RH, Brighton, UK

**Keywords:** experiment, stationary, crowd, model, collective cooperation, behavioural changes

## Abstract

Simulation models of pedestrian dynamics have become an invaluable tool for evacuation planning. Typically, crowds are assumed to stream unidirectionally towards a safe area. Simulated agents avoid collisions through mechanisms that belong to each individual, such as being repelled from each other by imaginary forces. But classic locomotion models fail when collective cooperation is called for, notably when an agent, say a first-aid attendant, needs to forge a path through a densely packed group. We present a controlled experiment to observe what happens when humans pass through a dense static crowd. We formulate and test hypotheses on salient phenomena. We discuss our observations in a psychological framework. We derive a model that incorporates: agents’ perception and cognitive processing of a situation that needs cooperation; selection from a portfolio of behaviours, such as being cooperative; and a suitable action, such as swapping places. Agents’ ability to successfully get through a dense crowd emerges as an effect of the psychological model.

## 1. Introduction

Simulation models of pedestrian dynamics are widely used today especially for evacuation planning [1–4]. Such models usually consist of unidirectional flows of agents (simulated pedestrians) and are used to estimate the evacuation time in emergency situations or to test safety concepts [5]. Simulations of such models are a useful tool in the planning phase to detect critical high densities for example to avoid casualties such as those reported at the Hajj on several occasions [6, p. 164] or at the Love Parade music festival 2010 in Germany [7].

**Figure 1.**
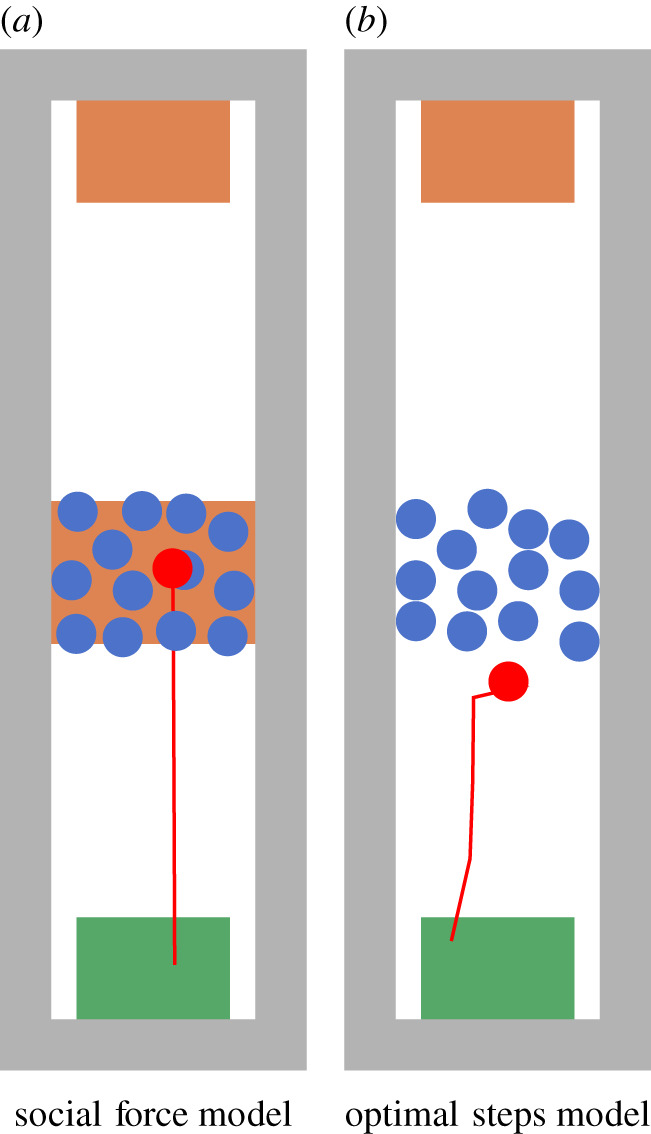
A walking agent (red) starts walking at the bottom area and tries to reach the rectangular target area on top while confronted with a dense, stationary crowd. In simulations, we cannot identify real interaction between agents when using different physically inspired locomotion models. Either the walking agent ‘ignores’ the dense, stationary crowd and walks on other agents which could not happen in real life (*a*). Or the crowd blocks the walking agent completely because of the high density (*b*). The open source simulator Vadere was used for the simulations. (*a*) Social force model and (*b*) optimal steps model.

Locomotion models [8–10] work well for unidirectional flows because they are mostly validated against empirical data [11]. They can provide helpful insights and make crowd gatherings safer. But often locomotion models fail for set-ups that seem only slightly different. For instance, when a first-aid attendant needs to forge a path through a dense crowd to reach an injured person. When re-enacting such a real-world situation in current simulation tools, agents often get stuck and end up in a deadlock situation because there is no real interaction between agents, compare figure 1.

### 1.1. Related work

Several authors extended existing locomotion models to manoeuvre agents through virtual environments and to mitigate shortcomings of these models. For instance, [12,13] let agents evade tangentially or sideways. Using such collision avoidance strategies on a microscopic level often leads to lane formation on a macroscopic level. But, pure physically inspired locomotion strategies like collision avoidance do not work for very dense crowds as seen by the simulations in figure 1. In the real world, humans adapt their behaviour [14, p. 11–12]. For instance, humans just ask to be let through. Humans use perception, cognition and a repertoire of different behaviours. [15] made first steps to integrate psychological findings into pedestrian dynamic simulations to control the social force model [9]. [15] integrated different agent states like queuing or pushing behaviour. [16,17] extended cellular automata to overcome deadlock situations in bidirectional pedestrian flows based on a more cooperative behaviour of agents. [16] integrated ideas from game theory and swerving preferences which are based on previous successful swerving behaviour. Xue *et al.* conducted their own experiment [18] and integrated a sort of ‘give way to counterflowing agents’ into a cellular automaton. However, both approaches were limited to cellular automata models, [16] even to one-dimensional scenarios. Also other simulator developers, both researchers and commercial ones [19,20], extended existing locomotion models to better cover waiting behaviour and other real-world situations. But all these extensions were integrated without providing empirical data or evidence. By contrast, [21] provided empirical evidence and developed a model to simulate crowd behaviour with social-cognitive agents with a focus on music festivals. They captured the motivation of individuals by including various physiological parameters like memory, bladder, stomach and arousal and goal-oriented agents. But adding a plethora of parameters on the individual level makes the model difficult to understand. Feliciani & Nishinari [22] extended a cellular automaton to allow greater densities and enabled swapping strategies for agents to maintain flow in counterflow scenarios. Nevertheless, to our knowledge there is no systematic operationalization of psychological processes to let agents pass through a stationary crowd. We argue that the classic locomotion models do not capture collective cooperation of real humans.

For us, the classic modelling process consists of making real-world observations and then finding mathematical and algorithmic formulations to describe the observed phenomena well. After implementing this as computer programs, we are able to carry out simulations to obtain further insights. In fact, another reason why models for high-density situations are still missing is the lack of empirical data. Numerous authors conducted experiments with a strong focus on unidirectional flow of pedestrians with moderate density [23–26] and counterflow scenarios [27,28] or bottlenecks [29,30]. Other authors focused more on collective phenomena in crowds. For instance, [31] investigated the influence of barriers on the behaviour of participants. They included the social psychology perspective by using questionnaires to obtain insights into participants’ perception. And other authors focused more on egress and queuing behaviour such as [32]. To our knowledge, [33] are the first authors who conducted an experiment with a stationary crowd and who tested the effects on walking participants. Even the exhaustive two-volume literature review [34,35] for empirical methods and experiments in pedestrian dynamics did not explicitly mention stationary crowds and their effects. So far, experiments have been conducted, but no model was derived or the model was described, but was not mapped to a clean and reusable software architecture. In fact, until recently, simulation frameworks for pedestrian dynamics completely lacked evidence-based models of cooperative actions, such as group actions [36]. Since then, first proofs of concept of specific situations have emerged, where empirical findings from social psychology, not analogy from physics, inspire the model. See [37,38]. Yet, to our knowledge, nobody has operationalized psychological findings into computer models of crowds where an agents’ ability to pass through a dense crowd emerges as an effect. We would like to close this gap.

But what should be the corner stone of such a psychological model? Prima facie, it seems that individuals manage to flow through dense crowds, and that this is achieved via cooperation from the crowd, who adjust themselves and move to give the individual a little space, rather than via force (since the latter would breach social norms around peaceful behaviour and politeness). For example, [26] show that when individuals approach a crowd in counterflow they do not simply walk into it nor do they simply stop but rather there is some negotiation of space among individuals to allow one to flow through the other. Thus, we argue that we need a model of crowd cooperation.

### 1.2. Goals of our work and article structure

In this contribution, we aim to model collective behaviour in a crowd so that the ability of agents to pass through dense static crowds emerges. Our goal is to directly base the model on empirical evidence and also to firmly put it into the frame of current social psychology. Finally, we strive for a reusable software structure and free and open-source implementation of the model that can be generalized to a large number of instances of cooperative collective behaviour.

The paper is structured as follows: In §2, we present a controlled experiment which we conducted in 2018 with students. We list our observations and formulate hypothesis which we test statistically. The most important, albeit almost trivial observation is, that the participants were indeed able to get through the crowd. In §3, we then describe the psychological processes in such a situation and operationalize it into a parsimonious model. That is, we restrict the model to elements that we deem absolutely necessary: the perception and subsequent cognition of a situation that calls for behavioural changes, the selection of a behaviour from a portfolio, notably being cooperative, and the selection of a suitable action, such as swapping positions. This operationalization represents a simple, generic and reusable model allowing more interactions between agents which can be easily implemented in different pedestrian simulation programs. We implement our model in a parsimonious computer program for which we run computer experiments in which the desired phenomenon emerges: agents are able to pass through a crowd. Finally, in §4, we evaluate our results. We present ideas how to add detail for a better quantitative match of second-order effects and we discuss how to generalize the model to encompass other collective phenomena.

## 2. Experiment

### 2.1. Experiment set-up

In order to study the effects of high densities on a walking person, we performed a controlled experiment in the foyer of the Munich University of Applied Sciences on 12 October 2019 (11.45–13.00).

In the experiment, we observed how a participant walks through a dense, waiting crowd. To this end, we kept 58 participants in a separate waiting room. The participants were entertained by experiment assistants with quizzes and discussions to keep the atmosphere as normal as possible. To avoid any priming, the participants received minimal information. The participants signed an informed consent form with the title ‘Study on movements of pedestrians’. The form stated that no physical risks were involved and that the experiment was recorded on camera. We chose first-year students in their second week as participants to ensure that they did not know anything about the experiment’s intentions.

**Figure 2.**
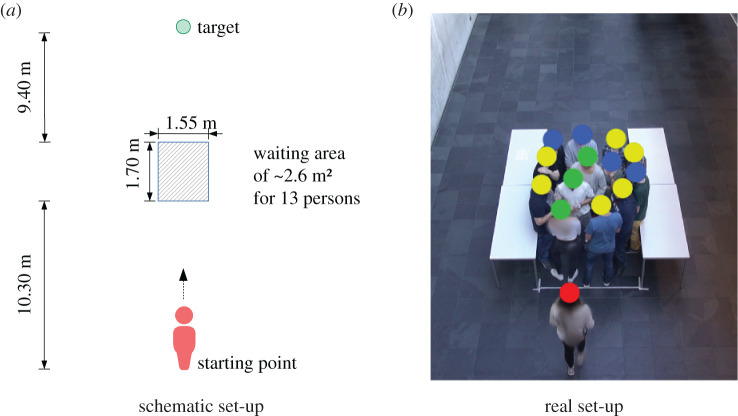
The experiment set-up: a waiting crowd of 13 participants in a delimited area of 2.64 m^2^ is successively crossed by a participant (figure from [39]). (*a*) Schematic set-up and (*b*) real set-up.

During the experiment, 13 participants stood in a delimited area of 2.64 m^2^ (1.55 m × 1.70 m) as a waiting crowd. In each experiment run, the walking participant successfully crossed the crowd along what would be the *y*-axis in figure 2*a*. The density while crossing was *ρ* = 5.30 ped m^−2^. For each experiment run, we randomly chose one person from the waiting room and assigned this person as walking participant. For the very first run, we also chose 13 persons from the waiting room and assigned them as waiting crowd.

We took two measures to avoid training effects for the waiting crowd: (1) after each run, a staff member shuffled the waiting crowd. To this end, the waiting crowd were asked to leave and re-enter the waiting area, so that the positions of the participants were shuffled. (2) After five runs, seven random participants of the waiting crowd were replaced by seven participants from the waiting room, who were also chosen randomly. We also took several measures to avoid observer biases like using a standardized experiment procedure with consistent instructions for all participants. The walking participants were instructed with the sentence ‘Go to the tree by crossing the crowd’. The waiting crowd was instructed with ‘Wait in the delimited area’. See [39] for a description of all measures. Tables on the left- and right-hand side of the waiting area prevented the participants from leaving the waiting area accidentally. The experiment set-up is depicted in figure 2 and described in more detail in [39].

**Figure 3.**
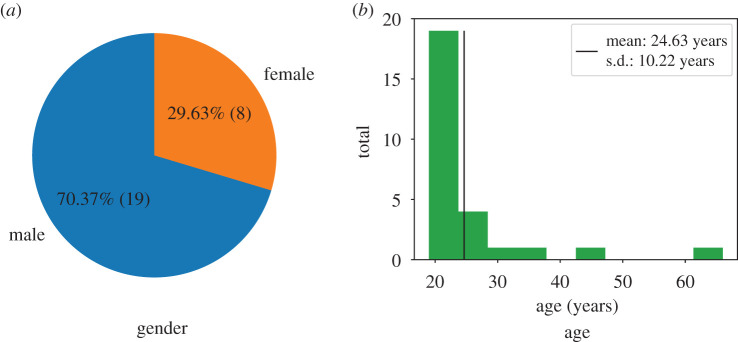
Gender and age distribution of the 27 walking experiment participants. (*a*) Gender and (*b*) age.

In total, 58 students participated in the experiment. Twenty-seven of them (men and women), aged 19–66, were assigned as walking participants and performed 30 runs (compare figure 3). We collected gender, age, height and shoulder width for each walking participant.

The experiment was filmed from above at an angle of around 60° (compare figure 2*b*). We recorded the experiment with a camcorder Sony Handycam HDR-PJ780VE using a resolution of 1280 pixel × 720 pixel and 25 frames per second. The raw video material had a length of 73 min. We used the free video analysis and modelling tool Tracker [40] to correct the optical distortion and to track the trajectories of the walking participant and the waiting crowd. For this purpose, we applied Tracker’s ‘Auto-Tracker’ feature. See electronic supplementary material, E1 for more information about trajectory extraction. After trajectory extraction, we used self-written Python scripts, more precisely Jupyter notebooks, to analyse the data.

### 2.2. Experiment results

We began by watching the experiment’s video footage. This step helped us to verbalize the human behaviour we observed and to formulate the following hypotheses:

— Pedestrians walking through a crowd are slowed down.
— The pedestrians in a waiting crowd return to their initial positions after giving way to the ‘intruder’.
— Real humans can pass a crowd at high densities.

The last hypothesis, while seemingly trivial, is the most important one, because this is where simulated agents have failed so far. In a second step, we will test these hypotheses and quantify effects.

#### 2.2.1. Experiment result: speed distributions

Firstly, we measured the instantaneous speed of the walking participant inside and outside the waiting crowd. Outside the crowd this gives an estimate of the ‘free-flow’ speed, which is the walking speed of a pedestrian if no external effects force the pedestrian to slow down or to speed up. We measured the ‘free-flow’ speed in front of the waiting crowd instead of behind because the area in front is closer to the camera and we expect a lower measurement error from optical distortion. The instantaneous speed *v*_*i*_(*t*) for walking participant *i* at time step *t* is defined as

2.1 $$v_{i}(t)=\frac{\sqrt{{\Delta x_{i}(t)}^{2}+{\Delta y_{i}(t)}^{2}}}{\Delta T},$$

where Δ*T* = 1/25 s = 0.04 s (that is, in the Tracker software we evaluated 25 camera frames per second) and Δ*x*_*i*_(*t*) = *x*_*i*_(*t*) − *x*_*i*_(*t* − 1).

Then we averaged the instantaneous speed values *v*_*i*_(*t*) over all time steps *N* when the participant was inside the measurement area

2.2 $${\bar{v}}_{i}=\frac{1}{N}\sum_{t=1}^{N}v_{i(t)}.$$

**Figure 4.**
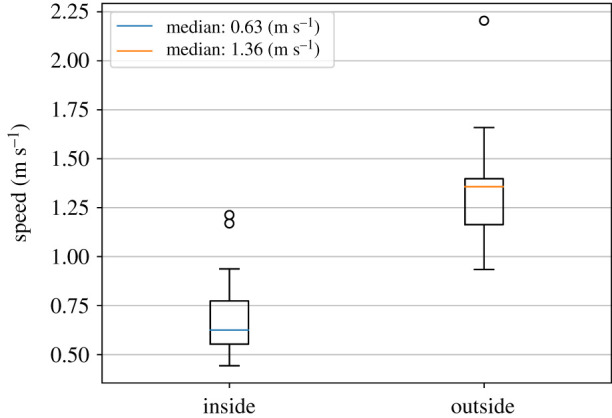
Box plot for speed distribution (averaged instantaneous speeds) of the walking participants inside and outside the waiting crowd.

Figure 4 and table 1 provide an overview of the averaged instantaneous speeds of all walking participants.

**Table 1.** Detailed statistics for the measured speed distributions of the walking participants inside and outside the waiting crowd.

	speed (m s^−1^)	
	inside	outside
sample size	30.00	30.00
mean	0.70	1.33
s.d.	0.19	0.25
min	0.44	0.93
25%	0.55	1.16
50%	0.63	1.36
75%	0.77	1.40
max	1.21	2.20

Comparing the mean instantaneous speed of 0.70 m s^−1^ (inside) and 1.33 m s^−1^ supports our hypothesis that the walking participants are slowed down by the waiting crowd. At a density *ρ* of 14 persons per 2.64 m^2^, that is, *ρ* = 5.30 persons/m^2^, we measure a ‘slow-down’ factor of 1.33/ 0.70 = 1.9 ≈ 2.

We also performed Student’s *t*-test to check if the waiting crowd has an effect on a walking participant’s speed. For this, we calculated for each walking participant *i*: Δ*v*_*i*_ = *v*_*i*,in_ − *v*_*i*,out_. Then, we applied a one-sided *t*-test with following mathematical hypotheses:

— *H*_0_ : *mean*(Δ*v*_*i*_) ≥ 0
— *H*_1_ : *mean*(Δ*v*_*i*_) < 0
— Significance level: 0.05

The test statistic $T=\sqrt{N}\times (\text{mean}(\Delta v_{i})-0)/\text{s.d.}(\Delta v_{i})$ revealed a value of *T* = −10.75 for all *N* = 30 participants. We drop the *H*_0_ hypothesis of no influence since our tests statistic *T* is far below the significance limit of 0.05 of the corresponding t distribution, which is −1.70 at a *p*-value ≪ 0.01.

When watching the video footage, we identified some potential outliers in the data. For instance, we observed a particular fast participant outside and inside the waiting crowd. The participant stretched out the hands like a swimmer to ‘dive’ through the crowd. We also observed a very slow participant inside the waiting crowd whom some members of the waiting crowd blocked intentionally. We decided to keep these outliers for our statistical analysis to stay close to the real world where one can also observe different techniques to cross a dense crowd. Some of these techniques are faster or slower than others.

The measured mean free-flow velocity of 1.33 m s^−1^ outside the waiting crowd is very close to previous empirical measurements like [41] with 1.34 m s^−1^. This strengthens our belief that we gathered realistic data.

#### 2.2.2. Experiment result: distribution of the waiting crowd

We want to shed light on the question of whether the waiting crowd participants return to their initial positions after giving way to an intruder. To analyse the movement of each participant of the waiting crowd, we looked at two metrics: first, we measured the Euclidean distance between the initial and the end position of each participant. Second, we looked at the maximum Euclidean distance a waiting participant walked. For this, we compared each position of a participant’s trajectory^1^ with the trajectory’s initial position.

**Figure 5.**
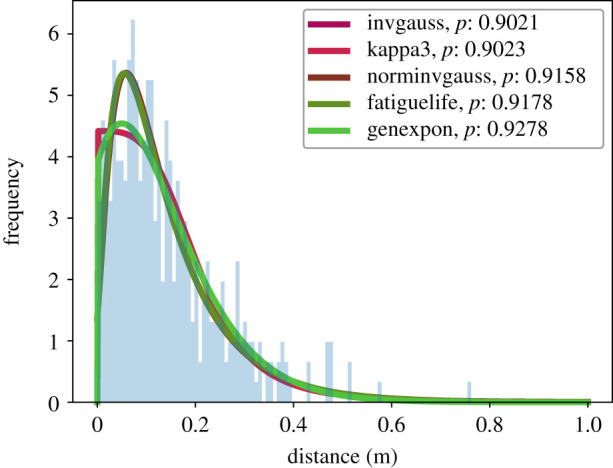
The data in blue visualizes the Euclidean distances between a participant’s initial position—before the walking participant entered the waiting crowd—and the end position. The Euclidean distance is defined as ||p initial−p end|| with $p\in R^{2}$. The plot includes the best-fitting continuous distributions with a *p*-value ≥0.90.

**Figure 6.**
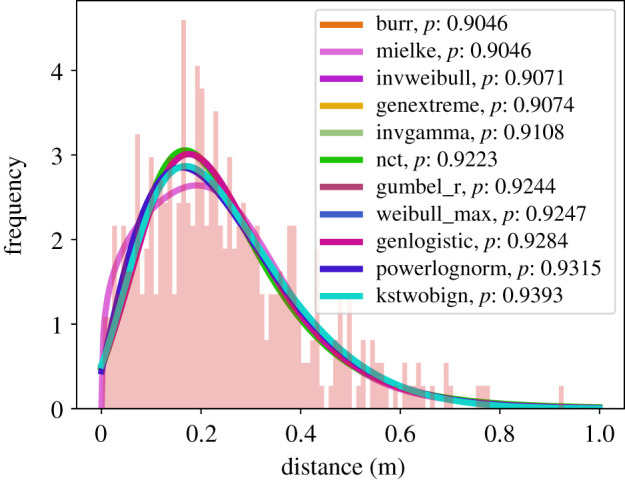
The data in red visualizes the maximum Euclidean distance a participant of the waiting crowd moved while the crowd was crossed by the walking participant. The plot includes the best-fitting continuous distributions with a *p*-value ≥0.90.

Then, we investigated if the extracted distances follow a continuous probability distribution. We tested the data against 94 distributions [42] and used the Kolmogorov–Smirnov test to verify the goodness of fit. The Kolmogorov–Smirnov test assumes as null hypothesis *H*_0_ that the sampled data and the tested distribution follow the same probability distribution. In our survey, we keep only distributions with a *p*-value greater 0.90. Figure 5 and table 2 summarize the data for the first metric (the Euclidean distance between initial and end position). Figure 6 and table 3 summarize the data for the second metric (the maximum Euclidean distance).

**Table 2.** Detailed statistics for the participants of the waiting crowd and the Euclidean distance between participant’s initial and end position.

	distances (m)
	(metric 1)
sample size	400.00
mean	0.14
s.d.	0.11
min	0.00
25%	0.06
50%	0.11
75%	0.19
max	0.76

**Table 3.** Detailed statistics for the participants of the waiting crowd and the maximum Euclidean distance

	distances [m]
	(metric 2)
sample size	400.00
mean	0.25
s.d.	0.16
min	0.00
25%	0.13
50%	0.22
75%	0.33
max	0.93

We cannot identify one single and best-fitting distribution for each of the two metrics. However, we observe that the best-fitting distributions are not of the same type for the two metrics. We also observe that the distribution of the maximum distance is broader, with a heavy tail towards a larger value.

We hypothesized that participants in the waiting crowd return to their initial positions. But, it would be unrealistic to expect them to hit the exact same spot. Also, people shift from one foot to the other which causes the head to sway for at least several centimetres which is also reported by [43, fig. 3, p. 4]. Thus, within an error margin, we would expect a distribution for the first metric, which is centred around a value, a little off from zero, by which waiting individuals, on average, miss their original position. This, in principle, is what we see. In any case, the participants do not stay at the position of maximum difference.

We argue that the data supports a tendency to return, where the mean distance from the initial position is only 0.14 m with a standard deviation of 0.1 m.

#### 2.2.3. Experiment result: trajectories and walking participant and duration in waiting area

**Figure 7.**
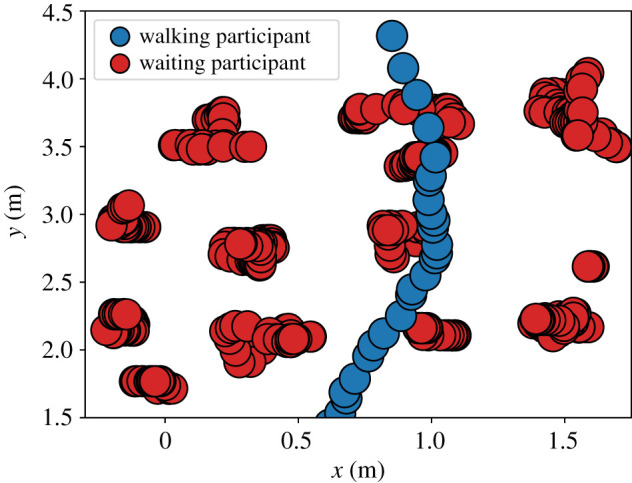
The trajectory of a single walking participant inside the waiting area at a time resolution of 1/25 s.

**Figure 8.**
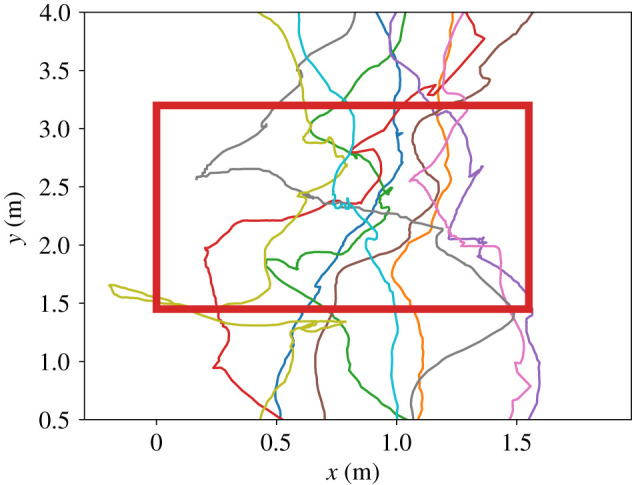
The trajectories of 10 walking participants inside the waiting area (red rectangle) at a time resolution of 1/25 s.

With a third set of measurements, we took a closer look at how walking participants manoeuvre through the waiting crowd. For this, we first plotted the trajectories of the walking participants, see figures 7 and 8.

**Figure 9.**
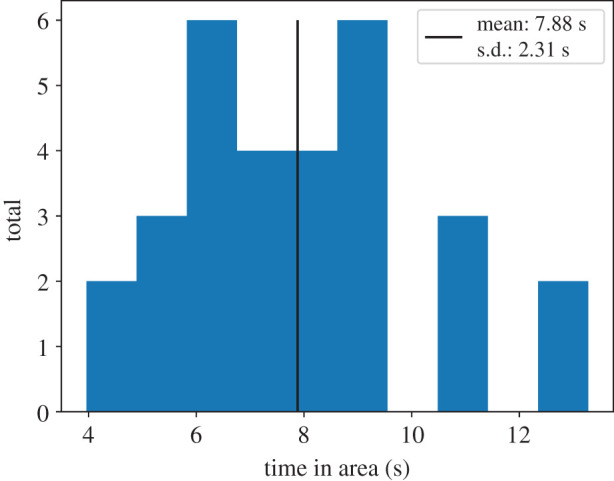
The duration of the walking participants inside the waiting area as histogram.

Then we measured the time the walking participants spent in the rectangular waiting area of 1.55 m × 1.7 m (width × height), see figure 9.

The trajectory plots show that **all** walking participants were able to cross the waiting crowd. Instead of straight lines, we observe curvy trajectories where walking participants move around a waiting person or both seem to swap places. Our measurements of the waiting participants’ maximum displacement in figures 6, and 7 show that the waiting participants also move. We argue that this indicates interaction. In fact, during the experiment we saw different techniques: communication through eye contact or asking verbally, but also shoving the waiting person aside. Recent virtual reality experiments that track eye-gaze in dense crowds underline pedestrians’ focus on the closest vicinity for interactions [44]. This supports our hypothesis that collaboration with the next neighbours enables pedestrians to navigate through a dense crowd. We will use this finding to choose a suitable action in our model of cooperative behaviour.

Figure 9 visualizes the duration a walking participant spends inside the waiting area. It indicates that the interaction process between the participants takes time. The mean duration of a walking participant’s stay inside the waiting area is 7.88 s. Note, that if a walking participant walked through the waiting area, on a straight line, with an instantaneous speed of 0.70 m s^−1^ (measurement from table 1), it would only take height/speed = 1.70 m/0.70 m s^−1^ = 2.43 s.

## 3. Model

### 3.1. The need for a psychology model complementing pure locomotion

**Figure 10.**
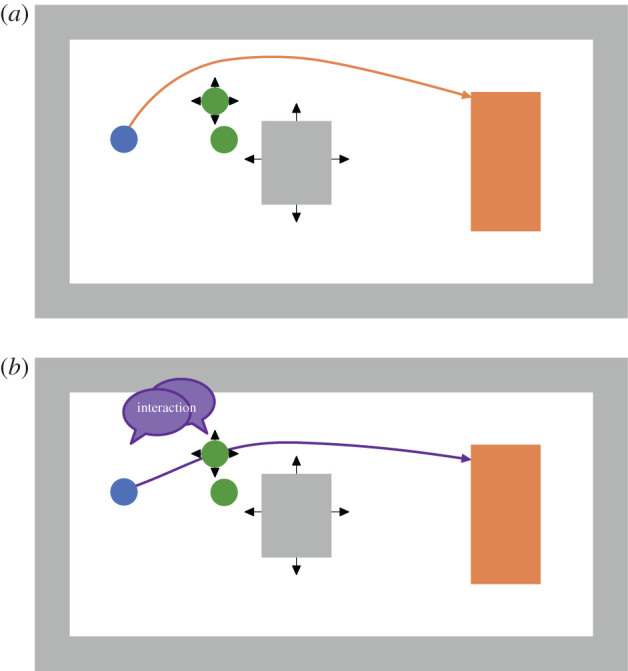
Current pedestrian stream models focus on a pure locomotion of agents and neglect scientific findings from psychology like interaction between people and collective actions in crowds exemplified by [36]. (*a*) Modelling only locomotion: a walking agent (blue) is repelled (black arrows) by other agents (green) and obstacles (grey) and attracted by its target (orange). The repulsion is visualized by arrows. (*b*) Modelling locomotion and psychology: a psychology layer allows agents to interact with each other.

In the experiment, **all** walking participants were able to cross the waiting crowd by interacting with the other participants. From this, we derived our hypothesis that real humans can pass a crowd at high densities. This simple hypothesis is essential since it is where pedestrian stream simulators often fail, see figure 1. We will focus on this challenge with our new model proposal. Attempts to solve the problem solely on the locomotion layer, through collision avoidance as depicted in figure 10 do not work for very dense crowds which is shown by the simulations in figure 1. In the real world, however, humans adapt their behaviour [14, pp. 11–12] to the situation. Humans use perception, cognition and a repertoire of different behaviours. They also interact.

We strive for a model that fulfils two important requirements: (1) firstly, the new model shall represent a generic architecture which can be easily integrated into different simulation tools, independent of the choice of locomotion model, and that can be generalized to other instances of collective cooperation. Thus, the new model will be beneficial for the whole research community. (2) Secondly, the new model shall be a faithful operationalization of psychological processes which affect the behaviour of agents. That is, it must be correct from a psychological perspective and it must be sufficiently simple to be understood by different research communities like computer scientists, physicists, sociologists or psychologists.

### 3.2. Model of a psychology layer for collective cooperation

Like for any other simulation software, a pedestrian stream simulator’s core is a simulation loop in which time is incremented. In this loop, a locomotion model is responsible for finding the next position for each agent in each simulated time step (compare electronic supplementary material, listing S1). Most of the current locomotion models [9,10,45,46] only include physical aspects to navigate an agent through an environment. For instance, obstacles repel an agent while targets attract agents.

But, the key is to include also the psychological status of an agent in each simulation step. This layer represents the mental processes of perception and cognition of real humans [14, p. 206ff.] and effects the behaviour of an agent. Additionally that means, instead of having just one behaviour—that is, moving towards a target—an agent must have a behavioural repertoire from which the agent can choose from to react to its environment. In the case of our experiment, that means that agents (both walking and waiting)

— on the perception sub-layer, perceive other agents in a sight/ search radius *r*;
— on the cognition sub-layer, realize that an agent cannot move anymore (that is, the speed over the last *n* steps is below a certain threshold) and they change their self-category [47] from target-oriented to cooperative to follow new social norms. We chose the term self-category here because [48, p. 20] states that ‘self-categorization [·· ·] becomes the psychological basis for crowd behaviour’ which is in our case collective cooperation;
— on the locomotion layer, being cooperative means that agents swap places to reach their target.

**Figure 11.**
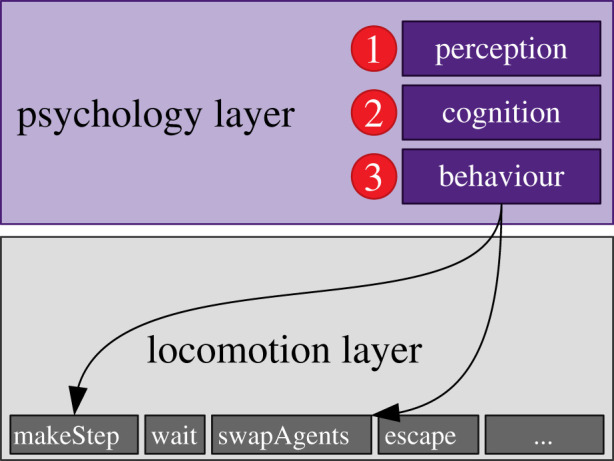
The three sequential phases of the new psychology layer: firstly, agents perceive environmental stimuli. Secondly, agents process this information in a cognition phase and enrich it with further (context-relevant) information. Thirdly, agents react to the processed information by selecting a behaviour from a behavioural repertoire on the locomotion layer. The behaviour repertoire on the locomotion layer should cover different real-world situations. For instance, make a step towards a target (e.g. a train station), wait at a platform (that is, do not move) or escape from a bang stimulus (which consists of several locomotion patterns).

Figure 11 visualizes the sequential processing of information inside the introduced psychology layer. The lower layers, e.g. cognition, process only the information from the direct upper layer. That means, an agent firstly perceives environmental stimuli, then an agent processes this information in the cognition layer and enriches it with further information (in case of the experiment this would be an agent’s speed). This simple architecture reflects what real humans do: perceive, process and react to this information with a specific behaviour. See figures in §3.3 to see the model in action and how target-oriented agents become cooperative and swap places to reach their target.

The main advantage of this clearly separated psychology layer is that experts in psychology or other fields can implement the perception and cognition sub-layers without knowing implementation details of the pedestrian stream simulator. A locomotion expert can implement the specific locomotion strategies. For instance, if cooperative behaviour does not mean swapping two agents, another locomotion strategy can be implemented on the locomotion layer. This clean software architecture makes it possible to work on a pedestrian stream simulator by combining knowledge from different research domains as proposed by [48, p. 46].

**Figure 12.**
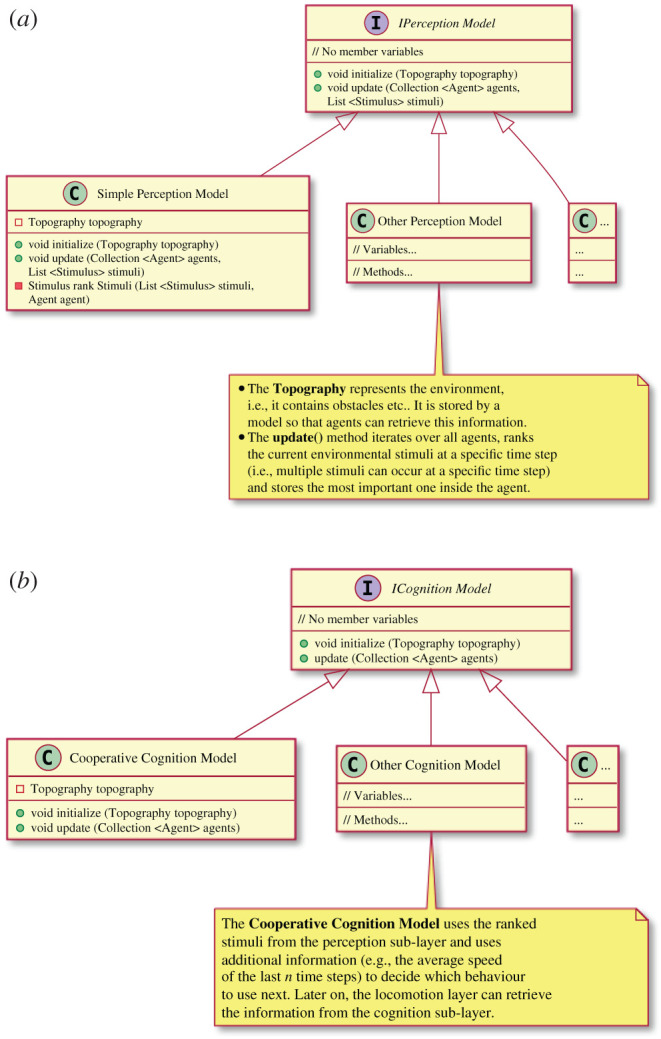
An UML diagram of the interfaces and classes of the perception and cognition sub-layers. Public methods are denoted with green circles, private methods are denoted with filled, red squares and member variables are denoted with unfilled, red squares. (*a*) Interfaces and classes of the perception sub-layer. (*b*) Interfaces and classes of the cognition sub-layer.

Introducing this psychology layer (with sub-layers perception, cognition and locomotion) modifies the existing simulation loop electronic supplementary material, listing S1 only very slightly and keeps the overall software architecture simple and easy to implement according to the KISS principle [49, p. 18; 50, p. 10], compare electronic supplementary material, listing S2. perceptionModel and cognitionModel are implementations of interfaces. Using this design decision—the strategy pattern—extends a pedestrian stream simulator to a tool that can also test psychological hypotheses. That means that it is possible to change the perception and cognition model for each simulation run and covers different real-world situations. For instance, an experiment situation differs from a daily commuting situation which affects humans’ perception and cognition. This reflects also the fact that a simulation tool cannot provide a ‘one-fits-all-situations’ model. Therefore, we facilitate interfaces with only two methods here, see UML diagram in figure 12.

Electronic supplementary material, listings S3 and S4 shows that it only requires 13 lines on the cognition sub-layer and 24 lines on the locomotion layer to obtain collective cooperative agents and to re-enact the experiment. For example, if an agent (walking and waiting) cannot move anymore, it becomes cooperative. Cooperative behaviour results in swapping positions. The proposed psychology layer was implemented in Vadere [51,52] because it is open source and has a well-validated locomotion layer [53,54]. Vadere is an open source framework for the simulation of microscopic pedestrian dynamics.

The following steps were carried out:

1. Add interfaces IPerceptionModel and ICognitionModel (see UML diagrams in figure 12).
2. To re-enact the experiment set-up from §2.1, implement SimplePerceptionModel and CooperativeCognitionModel. SimplePerceptionModel is empty because there were no external stimuli present in the experiment. CooperativeCognitionModel changes an agent’s self-category from target-oriented to cooperative if an agent cannot move anymore (that is, its speed is below a certain threshold by storing an agent’s psychology status with agent.setSelfCategory(SelfCategory newSelfCategory), see electronic supplementary material, listing S3.
3. Extend the existing simulation loop: In each simulation loop, invoke perceptionModel.update() and cognitionModel.update(), see electronic supplementary material, listing S2.
4. On the locomotion layer, evaluate agent.getSelfCategory() and react to it, see electronic supplementary material, listing S4.

### 3.3. Re-enacting the experiment with the collective cooperation model

**Figure 13.**
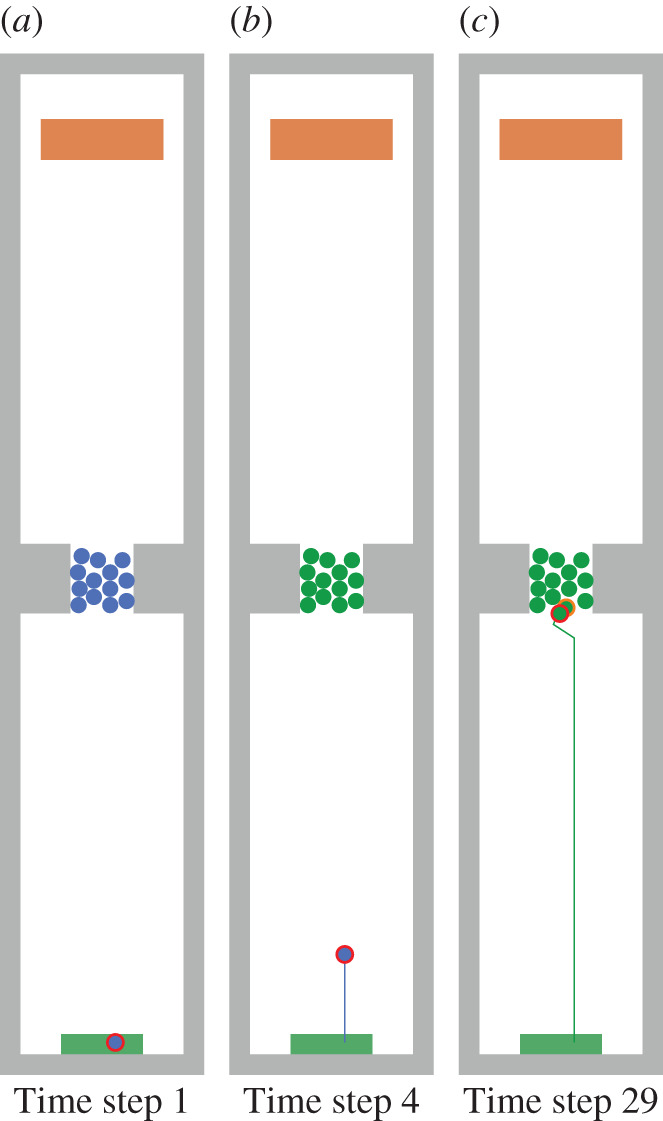
A walking agent (red-encircled) starts walking in the green source area and tries to reach the brown target area while the agent is blocked by a waiting crowd consisting of 13 agents. The colours represent the current behaviour of an agent: blue is target-oriented behaviour and green is cooperative behaviour. (*a*) Time step: when the simulation starts, all agents are target-oriented. While the walking agent is attracted by the brown target, the waiting crowd does not have a target and waits. (*b*) Time step 4: the agents of the waiting crowd become cooperative because their speed falls below a certain threshold. (*c*) Time step 29: the walking agent reaches the waiting crowd and cannot move anymore. Thus, the walking agent also becomes cooperative. The walking agent searches for a swap candidate (orange-encircled) and both swap positions.

**Figure 14.**
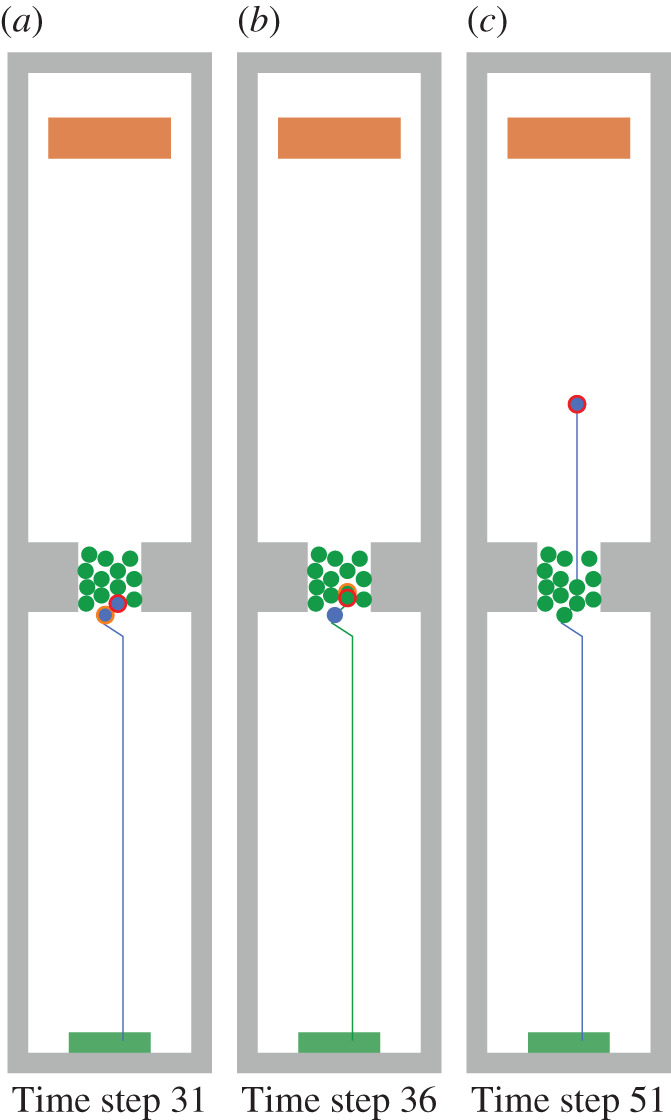
Cooperative behaviour of agents inside the waiting crowd. The colours represent the current behaviour of an agent: Blue is target-oriented behaviour and green is cooperative behaviour. (*a*) Time step 31: after swapping positions, the walking agent (red-encircled) and the swap candidate (orange-encircled) becomes target-oriented again because their speed is above a certain threshold. (*b*) Time step 36: the walking agent becomes cooperative again and swaps position with another cooperative agent, which is closer to the target. (*c*) Time step 51: the walking agent found its way through the dense crowd by using cooperative behaviour.

The psychology layer was implemented in the pedestrian stream simulator Vadere. We re-enacted the experiment set-up from §2.1 as closely as possible by using the same dimensions. We carried out 100 simulation runs with slightly varying initial position of the walking agent but consistent positions for the agents of the waiting crowd. Figures 13 and 14 show one of these simulation runs and visualize how the walking agent (red-encircled) changes its target-oriented behaviour to a cooperative one when the agent is blocked by the waiting crowd.

To validate the simulations, we compare the simulation results to the experiment results. In §2.2, we measured the speed of the walking participant, the spatial distribution of the waiting crowd and the trajectories of the walking participant. In our comparison, we omit the spatial distribution of the crowd because, in the implemented model, the agents of the waiting crowd just wait in the waiting area and do not move at all. This is what we assumed as—very simplified—waiting behaviour. That is, the travelled distance by the agents of the waiting crowd is zero. Therefore, it makes no sense to compare it with the experiment participants who moved continuously at least a bit.

**Figure 15.**
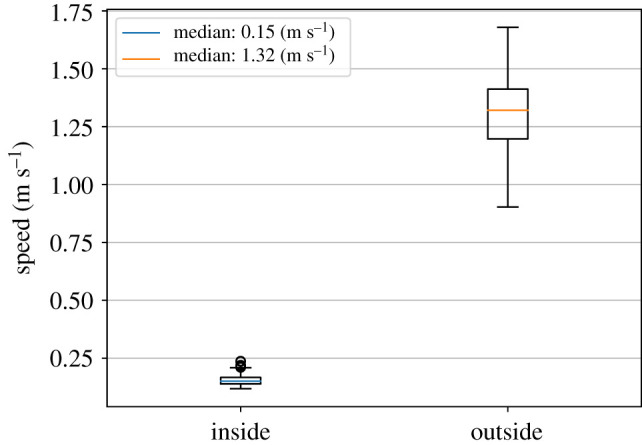
Box plot for speed distribution of the walking agent inside and outside the waiting crowd.

The 100 simulations reproduce the measured instantaneous ‘free-flow’ speeds at least qualitatively: the walking agents are slowed down inside the waiting area from 1.31 m s^−1^ (outside) to 0.16 m s^−1^ (inside) on average compared to 1.33 m s^−1^ and 0.70 m s^−1^ in the experiment, see figure 15 and table 4.

**Table 4.** Detailed statistics for the measured speed distributions of the walking participants inside and outside the waiting crowd.

	speed (m s^−1^)	
	inside	outside
sample size	100.00	100.00
mean	0.16	1.31
s.d.	0.03	0.15
min	0.12	0.90
25%	0.14	1.20
50%	0.15	1.32
75%	0.17	1.41
max	0.24	1.68

The speed of the walking agent inside the waiting crowd is much lower than what we have observed in the experiment. In the experiment, even if the walking participant is blocked by the waiting crowd for some moments, the walking participant constantly moves its body a tiny bit. That means the speed of the walking participant is constantly greater than zero. But in the simulation, it takes some simulation steps until a walking agent becomes cooperative when the agent is blocked by the waiting crowd. That is, the agent’s speed is zero for a lot of simulation steps, which lowers the average speed of the walking agents. Please keep in mind that this is the very first version of such a psychological model of collective cooperation and it will require some sort of calibration in the future.

**Figure 16.**
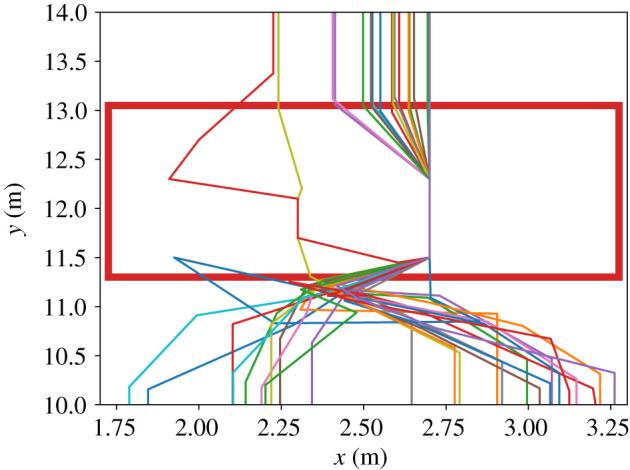
The trajectories of 25 walking agents inside the waiting area (red rectangle). Inside the waiting area, the walking agents follow zig-zag trajectories because they swap positions with agents of the waiting crowd. By changing to a cooperative behaviour, all walking agents were able to reach the target region. The agents of the waiting crowd are placed at the same positions for all 100 simulation runs. Therefore, we did not see a greater variety of the trajectories inside the waiting area.

**Figure 17.**
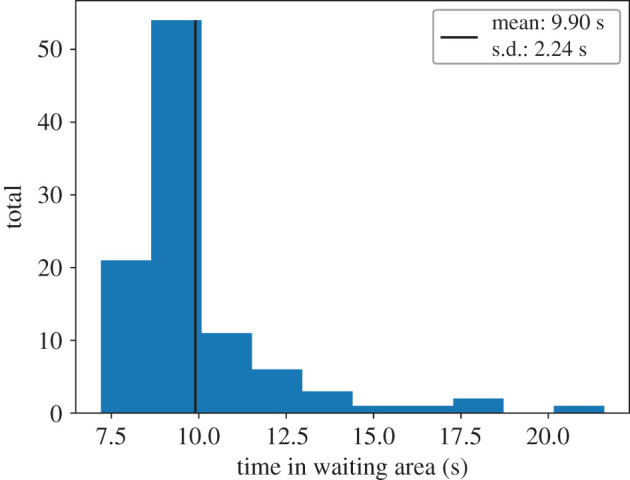
The duration of the walking agents inside the waiting area as a histogram.

Nevertheless, in our simulations, we see that **all** walking agents were able to cross the waiting crowd like in the experiment with real humans, see figure 16. Also the mean time of the walking agent inside the waiting area is very close to the experiment observations: 9.90 ± 2.24 s in simulation compared to 7.88 ± 2.31 s in the experiment, see figure 17.

## 4. Conclusion

We identified a major shortcoming in current pedestrian simulation models: the lack of collective cooperation which means that agents fail at seemingly simple tasks, such as forging a path through a dense crowd. Since empirical evidence is extremely scarce we also presented a controlled experiment to observe what really happens when participants pass a waiting crowd: we placed students in a delimited area of 2.64 m^2^ and let other participants walk through this waiting crowd. We took measures to avoid observer biases and to obtain reliable data from the experiment. We derived three hypotheses, namely: (1) real humans succeed in passing through a crowd at high density. (2) Pedestrians walking through a crowd are slowed down. (3) The pedestrians in the waiting crowd mostly return to their initial positions after giving way to the individual they allow through. While seemingly trivial, the first hypothesis is vital, because this is where classic locomotion models fail.

We presented a model where agents interact with each other to allow collective cooperation. Agents are able to perceive their environment, process this information and enrich it with additional information (within the simulation) in a cognition process. Then, agents select from a portfolio of possible behaviours, notably being target-oriented, or being cooperative. Actions on the locomotion layer follow, such as making a step towards a target or swapping place with a cooperation partner. The model is independent of the choice of locomotion model. It is implementation within the Vadere simulation framework is free and open-source.

In a re-enactment of the experiment situation, our simulations qualitatively reproduce the empirical observations. Most importantly, agents’ ability to pass through a dense crowd emerges as an effect of the psychological model.

In the future, we hope that new experiments and field observations will bring more qualitative and quantitative information on behaviours in dense crowds so that we—or other modellers—may add to the portfolio of behaviours, and calibrate parameters for a better quantitative fit. Furthermore, our generic approach covers a wide range of real-world situations of collective behaviours. As a next step, we would like to recreate people’s collective reaction to perceived threats within the same framework.

## Endnote

1 A trajectory encapsulates the positions of a participant over time.

## Data accessibility

We analysed the video material and extracted trajectories of all participants. These trajectories and scripts for analysis are placed in the ‘Pedestrian Dynamics Data Archive’ for public access: http://ped.fz-juelich.de/da/doku.php?id=motion_through_crowd (https://doi.org/10.34735/ped.2019.1).
